# Effect of the mitochondrial uncoupling agent BAM15 against the *Toxoplasma gondii* RH strain and Prugniaud strain

**DOI:** 10.1186/s13071-024-06187-8

**Published:** 2024-03-01

**Authors:** Zhendi Liu, Jiao Mo, Yetian Li, Siyang Liu, Qingyuan Zeng, Jili Zhang

**Affiliations:** 1 Health Science Center, Ningbo University, Ningbo, Zhengjiang Province, 315211 People’s Republic of China; 2 Lanzhou Institute of Husbandry and Pharmaceutical Sciences, Lanzhou, Gansu Province, 730050 People’s Republic of China

**Keywords:** BAM15, *Toxoplasma gondii*, Invasion, Proliferation, TEM

## Abstract

**Background:**

Toxoplasmosis is a zoonotic disease caused by the infection of the protozoa *Toxoplasma gondii (T. gondii)*, and safe and effective therapeutic drugs are lacking. Mitochondria, is an important organelle that maintains *T. gondii *survival, however, drugs targeting mitochondria are lacking.

**Methods:**

The cytotoxicity of BAM15 was detected by CCK-8 and the in vitro effects of BAM15 was detected by qPCR, plaque assay and flow cytometry. Furthermore, the ultrastructural changes of *T. gondii* after BAM15 treatment were observed by transmission electron microscopy, and further the mitochondrial membrane potential (ΔΨm), ATP level and reactive oxygen species (ROS) of *T. gondii *after BAM15 treatment were detected. The pharmacokinetic experiments and in vivo infection assays were performed in mice to determine the in vivo effect of BAM15.

**Results:**

BAM15 had excellent anti-*T. gondii* activity in vitro and in vivo with an EC50 value of 1.25 μM, while the IC50 of BAM15 in Vero cells was 27.07 μM. Notably, BAM15 significantly inhibited proliferation activity of *T. gondii *RH strain and Prugniaud strain (PRU), caused *T. gondii* death. Furthermore, BAM15 treatment induced *T. gondii* mitochondrial vacuolation and autolysis by TEM. Moreover, the decrease in ΔΨm and ATP level, as well as the increase in ROS production further confirmed the changes

**Conclusions:**

Our study identifies a useful *T. gondii* mitochondrial inhibitor, which may also serve as a leading molecule to develop therapeutic mitochondrial inhibitors in toxoplasmosis.’

**Graphical Abstract:**

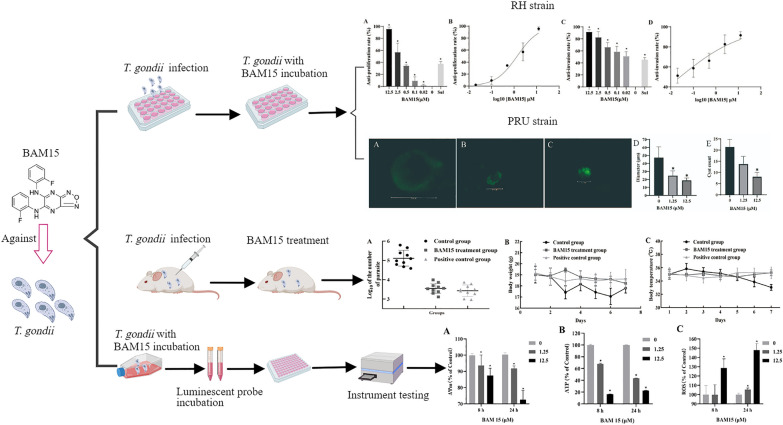

## Background

*Toxoplasma gondii* is an opportunistic pathogenic protozoan that is widely distributed worldwide and can cause toxoplasmosis in both humans and animals, seriously threatening human health [1]. *T. gondii* infection in immunocompetent individuals presents as a recessive infection with only mild flu-like symptoms occurring, while in immunocompromised persons, it causes serious consequences. Pregnant women infected with *T. gondii* for the first time may experience abortion, teratoma and stillbirth, and newborns may also suffer from congenital toxoplasmosis. Patients with organ transplantation and immunosuppressants are also prone to secondary toxoplasmosis [2, 3]. Additionally, *T. gondii* can cross the blood-brain barrier (BBB) via exploitation of DCs and monocytes as vehicles for their protection and transport, migrate and penetrate the brain or eyes, resulting in encephalitis (TE), neurological disorders, behavioral changes and eye disease [4]. Currently, drugs in acute toxoplasmosis are based on the combination of pyrimethamine and sulfadiazine, often accompanied by substantial bone marrow toxicity and ineffectiveness against chronic infection cysts of *T. gondii* [5, 6]. Therefore, future anti-*T. gondii* drugs with low toxicity and excellent effects on tachyzoites and cysts in toxoplasmosis have always been needed.

Currently, research on anti*-T. gondii* drugs mainly focuses on interfering with its folic acid metabolism, calcium signal transduction, type II fatty acid synthesis, DNA synthesis and energy metabolism [5]. Oxidative phosphorylation, a coupling reaction of ATP synthesis from ADP and inorganic phosphoric acid, supplied through the respiratory chain, is the main power source for *T. gondii* [7]. Some leading compounds targeting *T. gondii* mitochondria, such as atovaquone and endocrine-like quinolone (ELQ), were shown to have anti-*T. gondii* activity by targeting the *Q*_*i*_ or *Q*_*o*_ site of cytochrome b (a protein complex in the respiratory chain) [8]. Accordingly, mitochondrial oxidative phosphorylation is an attractive topic for the study of *anti-T. gondii* drugs.

BAM15, a mitochondrial uncoupler of oxidation and phosphorylation, is capable of transporting protons directly into the mitochondrial matrix without activating ATP synthase, thus avoiding oxidation reactions that produce ATP [9]. Moreover, BAM15 induced Ca^2+^ efflux from mitochondria to control oxidative stress and dysregulation of oxidative phosphorylation [10, 11]. Additionally, BAM15 can increase the rate of mitochondrial respiration by reducing the motility of protons that need to pass through the membrane inside the mitochondria while increasing the production of ROS [12]. Accordingly, the anti-*T. gondii* activity of BAM15 was investigated in vitro and in vivo under safe concentrations, and its mechanism of action was preliminarily explored.

## Methods

### Drugs, tachyzoites and cell culture

BAM15 (HY-110284, 99.26%) purchased from Med Chem Express (MCE, USA) was prepared as a 10 mM stock solution with dimethyl sulfoxide (DMSO, Sigma, USA). Vero cells were incubated with Dulbecco’s modified Eagle’s medium (DMEM), 100 U/ml penicillin, 100 μg/ml streptomycin, 1% nonessential amino acid (NEAA), 1% GlutaMax and 10% fetal bovine serum (FBS) at 37 °C in a 5% CO_2_ atmosphere. Type I RH strain tachyzoites and type II Prugniaud strain (PRU) of *T. gondii* used in our study were preserved in Vero cells in DMEM with 1% FBS, which was generously donated by the Lanzhou Veterinary Research Institute, China [13]. *Toxoplasma gondii* RH strain parasites, constitutively expressing nuclear green fluorescent protein (RH-GFP), were maintained in Vero cells.

### Cytotoxicity assay

Vero cells (1 × 10^4^ per well) were cultured in 96-well culture plates to monolayers and treated with BAM15 at final concentrations of 0.78125, 1.5625, 3.125, 6.25, 12.5, 25, 50 or 100 μM in DMEM with 1% FBS. Cells were incubated in DMEM with the equivalent of the maximum volume of DMSO used in drug treatments as a negative control. The blank control group without cells was only supplemented with DMEM containing 1% FBS. After 24 h of incubation, 10 μl of CCK-8 reagent (Biomake, USA) was added to each well for 1 h. Absorbance was detected at 450 nm with a Multiskan GO instrument (Thermo Fisher Scientific, MA, USA). Additionally, the cell survival rates were calculated with the formula: cell survival rate (%) = (absorbance of drug treatment group − absorbance of blank control group)/(absorbance of negative control group − absorbance of blank control group) × 100%. Triplicate independent experiments were performed.

### Plaque assay

The Vero cell monolayer was infected with 2 × 10^4^ *T. gondii* tachyzoites for 6 h and incubated with DMEM containing BAM15 (12.5, 2.5, 0.5, 0.1 or 0.02 μM) or DMEM without drug as a control. After 6 days of culture, the plaques were washed three times with PBS, and 4% paraformaldehyde was used to fix the plaques. Samples were stained with crystal violet for 10 min and then washed three times in PBS. After drying at room temperature, the number and size of the formed plaques were observed.

### Anti-invasion assessment of BAM15 on *T. gondii* RH strain tachyzoites in vitro

Vero cells were cultured to monolayers, incubated with DMEM containing different concentrations of BAM15 (12.5, 2.5, 0.5, 0.1 or 0.02 μM), sulfadiazine (1.6 μM, as a positive control) or without drugs in DMEM with 1% FBS as a control and then infected with 2 × 10^6^ fresh RH strain tachyzoites per well for 2 h. Then, the samples were washed twice with DPBS and then incubated with DMEM for 24 h. DNA was extracted, and the 529 bp repeat unit of *T. gondii* was detected as described in previous reports [13, 14]. Triplicate independent experiments were performed.

Vero cells were cultured to monolayers, incubated with DMEM containing different concentrations of BAM15 (12.5 and 1.25 μM), pyrimethamine (0.4 μM, as a positive control) or without drugs in DMEM with 1% FBS as a control and then infected with 2 × 10^6^ fresh RH-GFP strain tachyzoites per well for 2 h. Then, the samples were washed twice with DPBS and then incubated with DMEM for 48 h. Each group was observed by fluorescence microscope (Leica DM3000 LED, Germany) under 20 × and 40 × objective lens, and the number of PVs in each random field was counted by using LASx2D multi-channel analysis software, counting at least 10 random fields per well.

### Anti-proliferative activity of BAM15 on *T. gondii* RH strain tachyzoites in vitro

Vero cells were infected with 2 × 10^5^ tachyzoites per well. After 8 h, various concentrations of BAM15 (12.5, 2.5, 0.5, 0.1 or 0.02 μM) in DMEM with 1% FBS were added. DMEM without drugs was added for the control group, and 1.6 μM sulfadiazine was added under identical conditions as a positive control. After 24 h, the total DNA of the cell samples was extracted with DNAiso reagent, and the 529-bp repeat unit of *T. gondii* was detected by a QuantStudio 3 Flex Real-Time PCR System (Life Technologies), as described previously [15, 16]. The 50% effective concentration (EC_50_) of BAM15 that inhibited *T. gondii* growth was calculated. The results represent the mean ± standard deviation (SD) of at least three independent experiments.

### In vitro activity of BAM15 on *T. gondii* PRU strain tachyzoite and bradyzoite formation

Vero cell monolayers in 12-well plates were infected with 1 × 10^4^ PRU tachyzoites per well for 8 h, after which various concentrations of BAM15 (12.5, 2.5, 0.5, 0.1, 0.02 μM) in DMEM supplemented with 1% FBS were added to the cells. DMEM without drugs was added as a control. After 24, 48 or 72 h, the DMEM was removed, and the number of PRU strain parasitophorous vacuoles (PVs) was assessed by phase contrast microscopy (Olympus, CKX3, Japan) under a 40 × objective lens, and at least 20 random fields per well were counted.

For cyst formation of type II PRU strain, Vero cells grown on 12-well plates were infected with pre-processed parasites and cultured under bradyzoite-inducing conditions (DMEM medium supplemented with 50 mM HEPES and 1% FBS, pH 8.2, ambient CO_2_) for 3 days. The medium was removed and replaced by medium containing BAM15 (1.25 or 12.5 μM) or DMSO at 0.1% under the control condition. After 48 h, the DMEM was removed, and the effect on PRU strain bradyzoite cysts was assessed by fluorescence microscope (Leica DM3000 LED, Germany) under a 20 × objective lens. The number and size of all cysts in each sample were counted and measured. The images were analyzed using LASx2D multi-channel analysis software.

### Flow cytometry analysis

For RH strain tachyzoites, approximately 1 × 10^6^ extracellular RH strain tachyzoites were respectively incubated with BAM15 (1.25 or 12.5 µM) in DMEM or with no drug as a control for 8 h, followed by centrifugation at 4000 rpm for 10 min. The samples were washed with PBS and suspended in 100 µl binding buffer with 5 µl Annexin V-PE and 5 µl FITC dye in the dark for 15 min at room temperature. Double mixtures were acquired and analyzed by flow cytometry (CytExpert, USA) [17]. The experiment was repeated three times.

For PRU strain tachyzoites, Vero cells were seeded in 25 cm^2^ culture flasks and infected with PRU strain tachyzoites. After 24 h of infection, the samples were treated with BAM15 (1.25 or 12.5 µM) or untreated for 24 h as a control. Approximately 1 × 10^6^ intracellular *T. gondii* PRU tachyzoites were extracted, followed by centrifugation at 4000 rpm for 10 min. The samples were washed with PBS and suspended in 100 ul binding buffer with 5 ul Annexin V-PE and 5 ul FITC dye in the dark for 15 min at room temperature. Double mixtures were acquired and analyzed by flow cytometry (CytExpert, USA) [17]. The experiment was repeated three times.

### Transmission electron microscopy (TEM) analysis

The Vero cell monolayer was infected with 2 × 10^6^ tachyzoites per well for 8 h, and the cells were cultured in DMEM containing 1.25 BAM15 for 8 h and 24 h, washed twice with PBS, fixed at room temperature for 1.5 h with 2.5% glutaraldehyde solution, fixed overnight at 4 °C and washed three times with PBS. The samples were fixed with 1% osmic acid solution for 1.5 h and washed three times with PBS. Then, the samples were eluted with gradient ethanol solution and 100% ethanol twice and embedded in Epon. After BAM15 treatment, the samples were ultrathin sectioned and observed with transmission electron microscopy (Tecnai Spirit Bio-TWIN, Thermo, FEI, USA) [18].

### Mitochondrial membrane potential (Δ*Ψm*) in *T. gondii* tachyzoites

*T. gondii* tachyzoites in Vero cells were incubated with BAM15 in DMEM for 8 h and 24 h; then, fresh *T. gondii* tachyzoites from Vero cell were isolated and purified. Approximately 1 × 10^6^ RH strain tachyzoites were washed with PBS and co-incubated with the MitoTracker Red CMXRos probe (50 nM, Invitrogen, USA) for 20 min. The fluorescence intensity was observed by laser confocal microscopy (LEICA TCS SP8, Germany) and analyzed by LASx2D multi-channel analysis software [16].

### Adenosine triphosphate (ATP) level in *T. gondii* tachyzoites

*T. gondii* tachyzoites (1 × 10^6^/each sample) in Vero cells were incubated in DMEM with BAM15 for 8 h and 24 h; then, fresh *T. gondii* tachyzoites from Vero cells were isolated and purified with 200 μl of lysis buffer. The lysates were then collected and incubated at 12,000 × g for 5 min, and 20 μl of the supernatants was added to each well containing 100 μl of ATP detection working dilution provided by the kit (S0027, Beyotime Biotech, Inc.). Then, the luminescence of each well was detected at 25 °C using a multilabel reader. The ATP level was calculated according to the ATP standard curve established by gradient dilution of the ATP standard provided by the kit (S0027, Beyotime Biotech, Inc.), and the results are expressed as μmol (μM). Three independent experiments were performed [16].

### ROS production assay

*T. gondii* tachyzoites (1 × 10^6^/sample) in Vero cells were treated with DMEM containing BAM15 or without drug as a control, and fresh 2 × 10^6^ *T. gondii* tachyzoites were seeded into black 96-well microplates, washed with PBS twice and then co-incubated with 7.5 μM CMH2DCFDA (Solarbio, D6470) for 10 min. After incubation, *T. gondii* tachyzoites were washed three times with PBS to remove excess probe. *Toxoplasma gondii* tachyzoites were suspended in 100 μl/well PBS, and the fluorescence intensity was measured by a Tecan Infinites M200 microplate reader. The excitation and emission filters were 495 ± 9 nm and 530 ± 20 nm, respectively [19].

### Pharmacokinetics of BAM15 in mouse plasma

BAM15 was dissolved in saline with 10% ethanol and 10% Cremophor EL at a concentration of 1 mg/ml. BAM15 was then injected intraperitoneally into nine BALB/c female mice at a dose of 10 mg/kg. Blood samples (100 μl) were collected at 0 h, 0.083 h, 0.25 h, 0.5 h, 1 h, 2 h, 4 h, 8 h, 12 h and 24 h. After all blood samples had been centrifuged at 3000 rpm for 10 min, plasma samples were collected and immediately stored at − 20 °C until analysis. Then, BAM15 levels in mouse plasma were determined by high-performance liquid chromatography-tandem mass spectrometry (HPLC-MS/MS).

Pharmacokinetic parameters were calculated using WinNonlin professional software version 8.0 (Pharsight, Mountain View, CA, USA). The optimal pharmacokinetic model was determined according to the principle of the minimum Akaike information criterion (AIC) value and used for data fitting and parameter estimation. The area under the plasma curve (AUC), peak plasma concentration (*C*_max_), half-life (*T*_1/2_) and peak time (*T*_max_) are expressed as the mean ± SD [20].

### In vivo activity of BAM15 against *T. gondii* RH strain tachyzoites

BALB/c mice were infected intraperitoneally with 1 × 10^3^ *T. gondii* RH strain tachyzoites for 4 h and then treated with BAM15 (10 mg/kg·bw, intraperitoneal injection); the positive control drugs (100 mg/kg·bw sulfadiazine, 50 mg/kg·bw pyrimethamine or 15 mg/kg·bw folinic acid, oral administration) or PBS (as a control). Each group was treated once a day for 7 consecutive days. One milliliter of mouse ascites was collected, and total DNA was extracted. The number of parasites in each sample was detected by RT-PCR as previously described [19]. The experimental procedure was approved by the institutional ethics committee of the Lanzhou Institute of Husbandry and Pharmaceutical Sciences, China.

### Statistical analysis of the data

Data were compiled by SPSS 23.0 (SPSS, Inc.; Chicago, IL, USA) and GraphPad Prism 6 (San Diego, CA) software. Data are presented as the mean ± SD. Independent Student’s t-test was used for the comparison of two groups. When the data are greater than or equal to three groups, one-way or two-way analysis of variance (ANOVA) with Tukey’s multiple comparison was conducted for data that fit the normal distribution, while one-way ANOVA with Dunnett’s multiple comparison was tested for data with small sizes or abnormal distributions. *p* < 0.01 was considered significantly.

## Results

### Cytotoxic activity

**Fig. 1:**
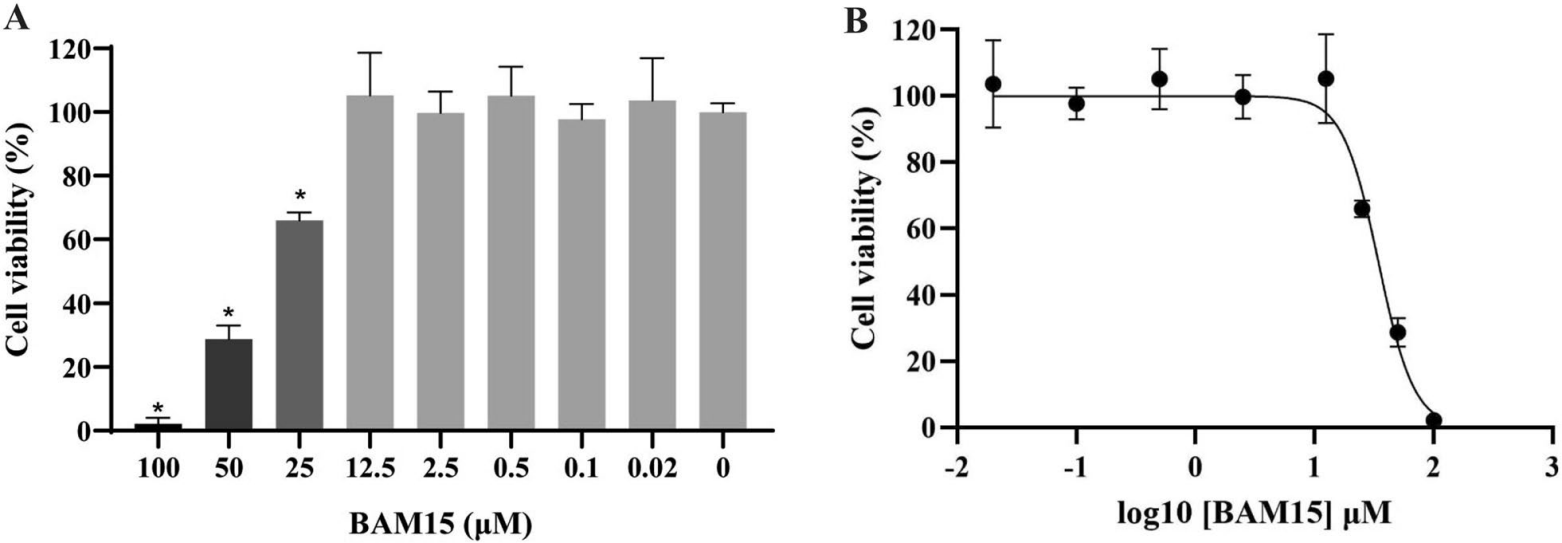
**A** Cytotoxicity of BAM15 on Vero cells. **B** Dose-response curve of BAM15 on Vero cells. All data are expressed as the mean ± standard deviation (SD), and the experiment was repeated three times. Compared with the control group, *p < 0.01

A cell viability assay was used to evaluate the safe range of BAM15 for host cells. BAM15 inhibited cell growth in a dose-dependent manner, showing no toxicity to Vero cells in the concentration range of 0–12.5 μM, and then its toxicity increased with the increase of the concentration, with an IC_50_ value of 27.07 μM (Fig. 1b). The maximum tolerated dose (cell viability rate ≥ 100%) of BAM15 to host cells was 12.5 μM (Fig. 1a).

### Anti-*T. gondii* tachyzoite proliferation and invasion

**Fig. 2:**
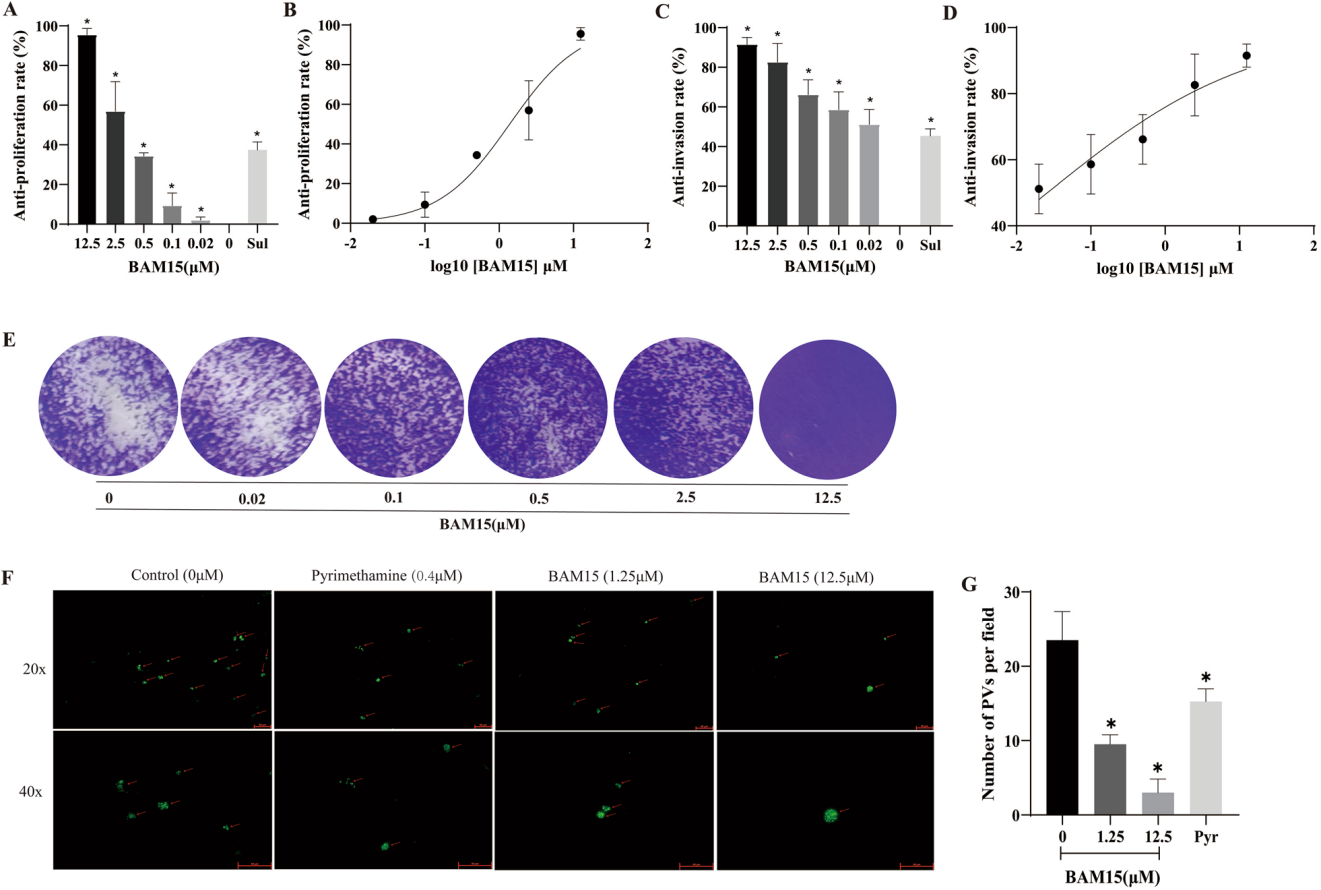
Anti-proliferative and anti-invasion effect of BAM15 on *Toxoplasma gondii*. **A** Anti-proliferative effect of BAM15 on *T. gondii*-infected Vero cells was detected by qPCR. The inhibition rate of tachyzoite proliferation in each group was compared with that in the control group. **B** Dose-response curve of BAM15 against *T. gondii* proliferation. Data are presented as the mean ± SD of three independent experiments. *p < 0.01 compared with the control group. **C** Anti-invasion rate of BAM15 in *T. gondii*-infected Vero cells. **D** Dose-response curve of BAM15 against *T. gondii* invasion. Three independent experiments were performed, and the data are expressed as the mean ± SD. *p < 0.01 compared with the control group. **E** Plaques formed by *T. gondii*-infected cells after BAM15 treatment. BAM15 (12.5, 2.5, 0.5, 0.1, 0.02 or 0 μM) decreased *T. gondii* growth inhibition in Vero cells. **F**, **G** Anti-invasion effect of BAM15 was observed by fluorescence microscope. *T. gondii* RH-GFP strain was treated with BAM15 (1.25 or 12.5 μM), pyrimethamine or without drug as a control for 2 h before infecting Vero cells. Scale bars: 50 μm

BAM15-mediated inhibition of the intracellular proliferation of *T. gondii* tachyzoites was detected by qPCR. Compared with the control group, BAM15 effectively inhibited the intracellular proliferation of *T. gondii* tachyzoites in a dose-dependent manner with an EC_50_ value of 1.25 μM (Fig. 2a, b), and the therapeutic index was 21.66. As a positive control drug, sulfadiazine inhibited the intracellular proliferation of *T. gondii* tachyzoites by 37.50%.

In the anti-invasion assay, compared with the control group, BAM15 also obviously suppressed the invasion of *T. gondii* in a concentration-dependent manner (*p* < 0.01), with an EC_50_ value of 0.025 μM (Fig. 2c, d). The rate of inhibition of sulfadiazine on invasion was 45.37%. In addition, the results of fluorescence microscopy were consistent with those of qPCR assay. Compared with the control group and positive drugs, the number of PV formed by *T. gondii* invasion was significantly reduced after treatment with BAM15 (Fig. 2f, g). Therefore, BAM15 significantly decreased the invasion of *T. gondii*.

To directly observe the growth inhibition of *T. gondii* by BAM15, we performed a plaque formation test. The size and number of plaques were affected by the concentration of BAM15 (Fig. 2e). The plaques were reduced in a dose-dependent manner in the range of 0.02–12.5 μM, demonstrating the inhibitory effect of BAM15 on *T. gondii*.

### BAM15 inhibited *T. gondii* PRU strain tachyzoite growth and cysts formation in vitro

**Fig. 3:**
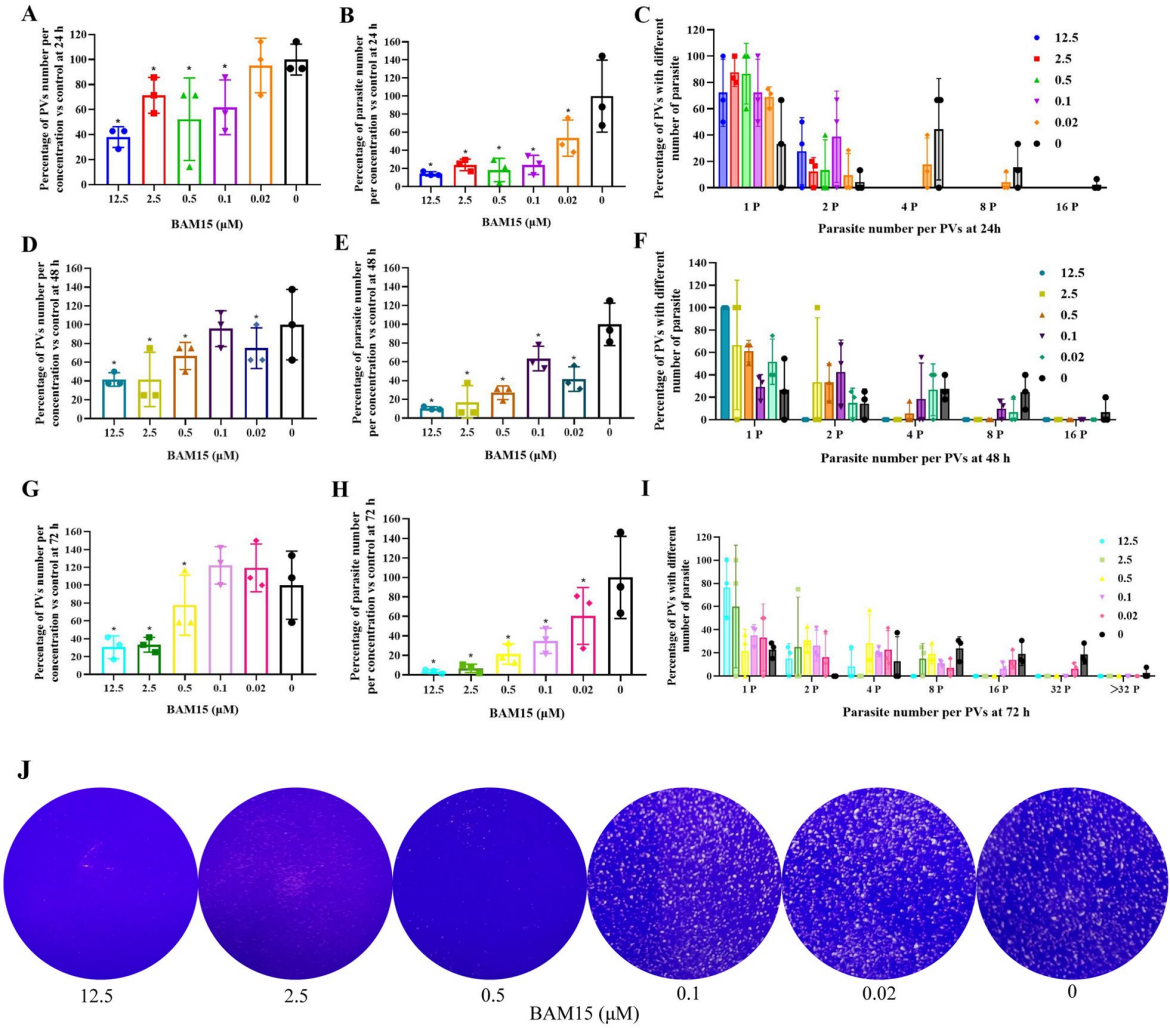
BAM15 decreased the number of PVs and parasites in PV of *T. gondii* PRU strain after 24 h, 48 h, 72 h incubation, as shown in **A**–**J**. ^*^P ≤ 0.01 compared with the control at 24 h, 48 h, 72 h incubation

Figure 3 shows that BAM15 significantly inhibited proliferation ability of *T. gondii* PRU strain tachyzoites after 24 h, 48 h and 72 h of incubation (*P* ≤ 0.01). Accordingly, compared with the control groups, the PV number significantly decreased after BAM15 treatment (Fig. 3a, d, g, *P* ≤ 0.01) in a time-dependent manner, and the average number of PRU strains within the PVs was also decreased (Fig. 3b, e, h, *P* ≤ 0.01). By comparison, the number of parasites per PVs was significantly decreased in a dose- and time-dependent manner, as shown in Fig. 3c, f, i. The plaque assay once again confirmed the inhibitory effect (Fig. 3j).

**Fig. 4:**
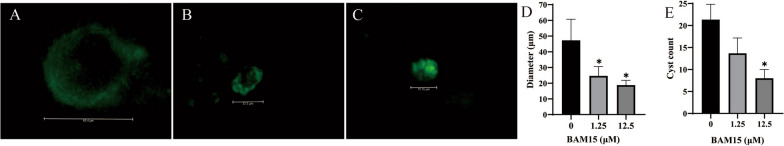
Changes in the shape, size and number of *T. gondii* PRU strain bradyzoite cysts after BAM15 treatment, as shown in **A**–**E**

Cysts of PRU strain bradyzoites formed in Vero cells in vitro, as shown in Fig. 4. BAM15 could inhibit cyst formation. Compared with the control group (Fig. 4a), the size of cysts was significantly reduced, and the shape of cysts was changed markedly, as shown in Fig. 4b or c. After treatment with 1.25 or 12.5 μM BAM15, compared to the control group, the size of cysts was reduced by 40% and 54%, respectively, as shown in Fig. 4d. Meanwhile, BAM15 significantly reduced the number of cysts at 12.5 μM concentration compared to the control group, as shown in Fig. 4e.

### BAM15 induced *T. gondii* RH strain and PRU strain tachyzoite death

**Fig. 5:**
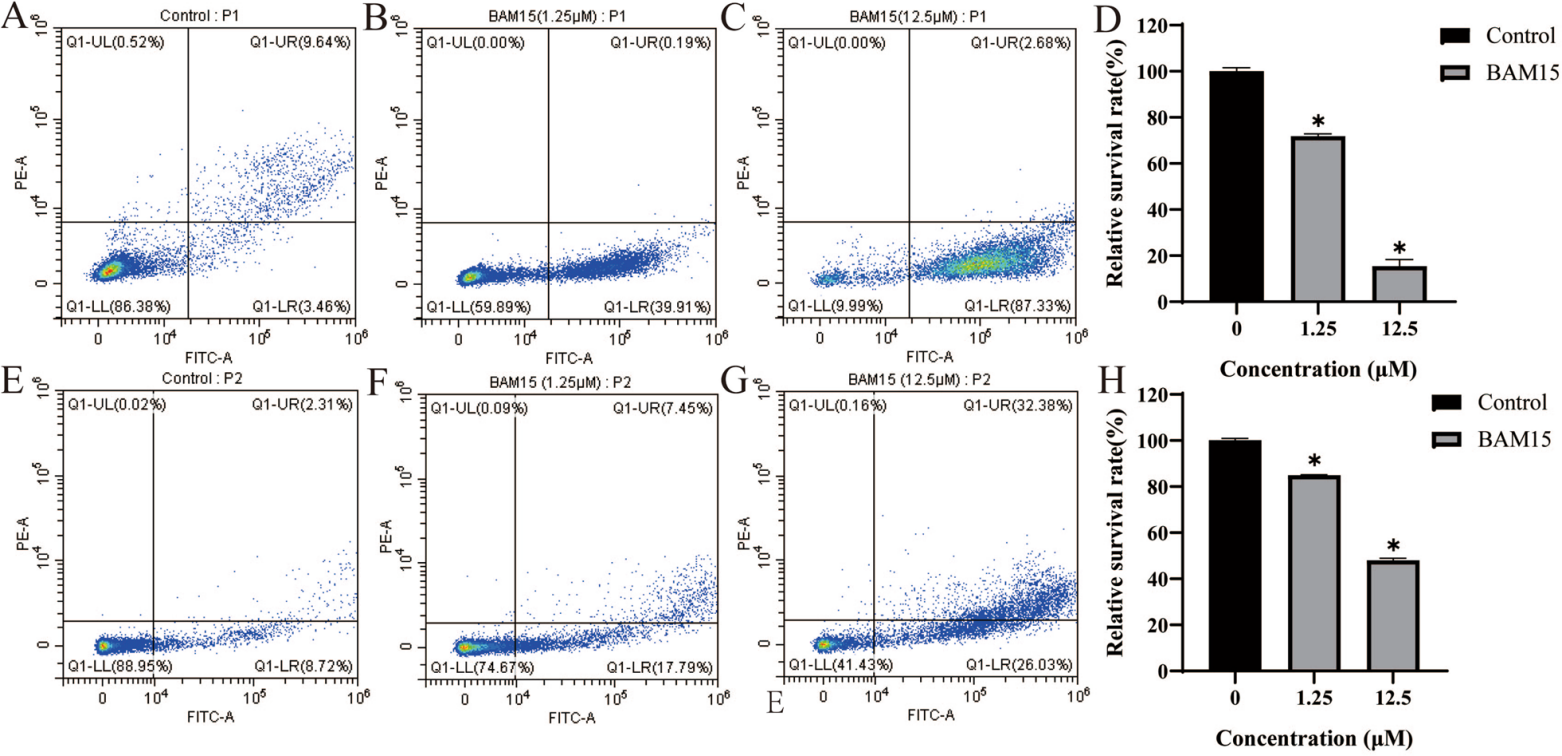
Flow cytometry analysis in BAM15 induced death in *T. gondii* RH strain and PRU strain tachyzoites Q1-LL: Lower left quadrant, Normal living cells (FITC-/PE-); Q1-LR: Lower right quadrant, Early apoptotic cells (FITC+/PE-); Q1-UR: Upper right quadrant, Middle and late apoptotic cells (FITC+/PE+); Q1-UL: Upper left quadrant, Necrotic cells (FITC-/PE+).’

To determine whether treatment with BAM15 was correlated with *T. gondii* RH strain and PRU strain tachyzoite death, the parasite survival rate was monitored by flow cytometry analysis. As shown in Fig. 5, BAM15 treatment significantly (*p* < 0.01) reduced *T. gondii* RH strain tachyzoite and PRU strain tachyzoite survival.

After treatment with 1.25 or 12.5 μM BAM15 for 8 h, the survival rates of RH strain tachyzoites were approximately 60% and 10%, respectively (Fig. 5b, c). Conversely, the control group of *T. gondii* RH strain tachyzoites showed a survival rate of approximately 86% (Fig. 5a). The relative proportions of *T. gondii* RH strain tachyzoites surviving after treatment with BAM15 (1.25 or 12.5 μM) or not for 8 h, respectively, are shown in Fig. 5d.

Compared with the control group of PRU strain tachyzoites (Fig. 5e), the survival rates of PRU strain tachyzoites were significantly decrease after treatment with 1.25 or 12.5 μM BAM15 for 24 h, as indicated by rates of approximately 75% and 40%, respectively (Fig. 5f, g). The relative proportions of *T. gondii* PRU strain tachyzoites surviving after treatment with BAM15 (1.25 or 12.5 μM) or not for 24 h, respectively, are shown in Fig. 5h.

### Ultrastructural changes in *T. gondii* tachyzoites after BAM15 treatment

**Fig. 6:**
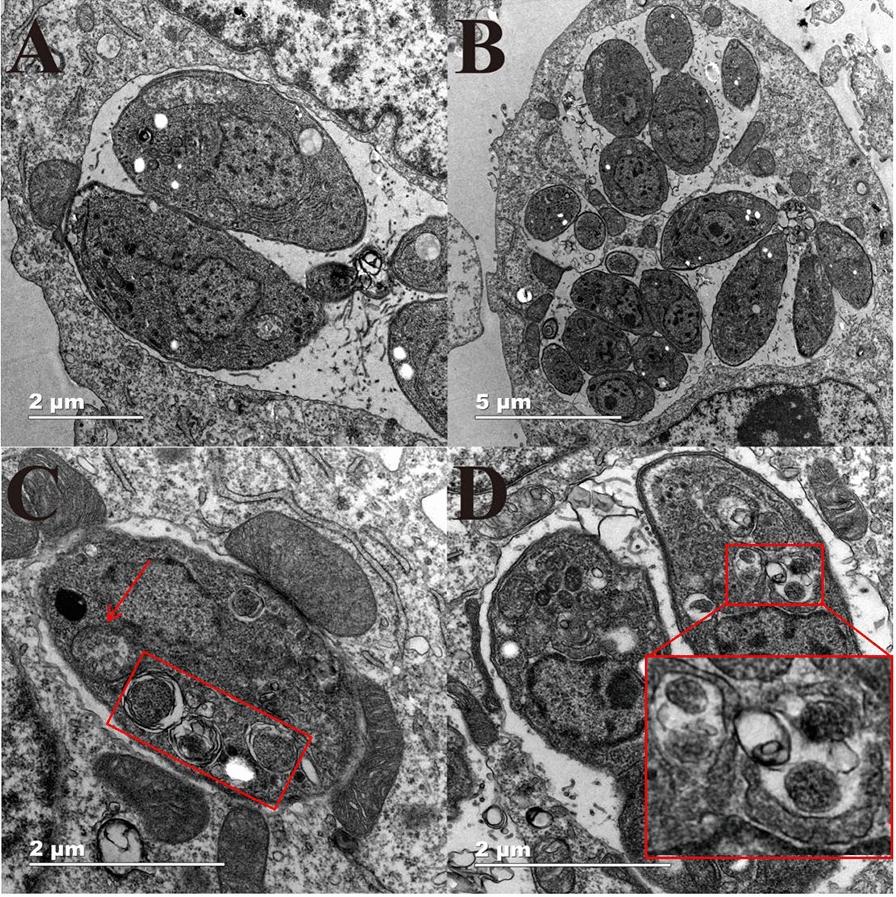
Ultrastructural changes in *T. gondii*-infected Vero cells with or without BAM15 treatment. *T. gondii* in the control group proliferated in the form of endodyogeny within PVs (**A, B**). After 8 or 24 h, BAM15 treatment induced tachyzoite mitochondrial vacuolation and autolysis, as shown in **C**, **D**. The red arrows indicate swollen, denatured mitochondria, and the red boxes indicate *T. gondii* vacuolation. Scale bars: 2 μm (**A, C, D**); 5 μm (**B**)

Ultrastructural changes in parasites were observed by TEM, which showed that the main organelle targeted by BAM15 was the mitochondrion. *T. gondii* in the control group proliferated in the form of endodyogeny within PVs surrounded by host cell mitochondria. The typical structures of *T. gondii*, micronemes, conoids and rhoptries were clearly visible (Fig. 6a). Banana-like parasite proliferation in PVs was observed in the control group after 24 h (Fig. 6b). After incubation with BAM15 (1.25 μM) for 8 h, the interior ultrastructure of parasites was full of numerous vacuoles, and the structure arrangement was disorganized. Therefore, mitochondria exhibited progressive degeneration and a swollen morphology, yet the membrane was still intact (Fig. 6c). After 24 h of incubation with BAM15 (1.25 μM), the shape of the *T. gondii* tachyzoites was distorted overall with absolutely disordered internal organelles and barely distinguished mitochondrial morphology. More interestingly, host cell mitochondria demonstrated normal conditions (Fig. 6d). These results suggested that the effects of BAM15 against *T. gondii* may be associated with mitochondria.

### Effect of BAM15 on mitochondria of *T. gondii* tachyzoites

**Fig. 7:**
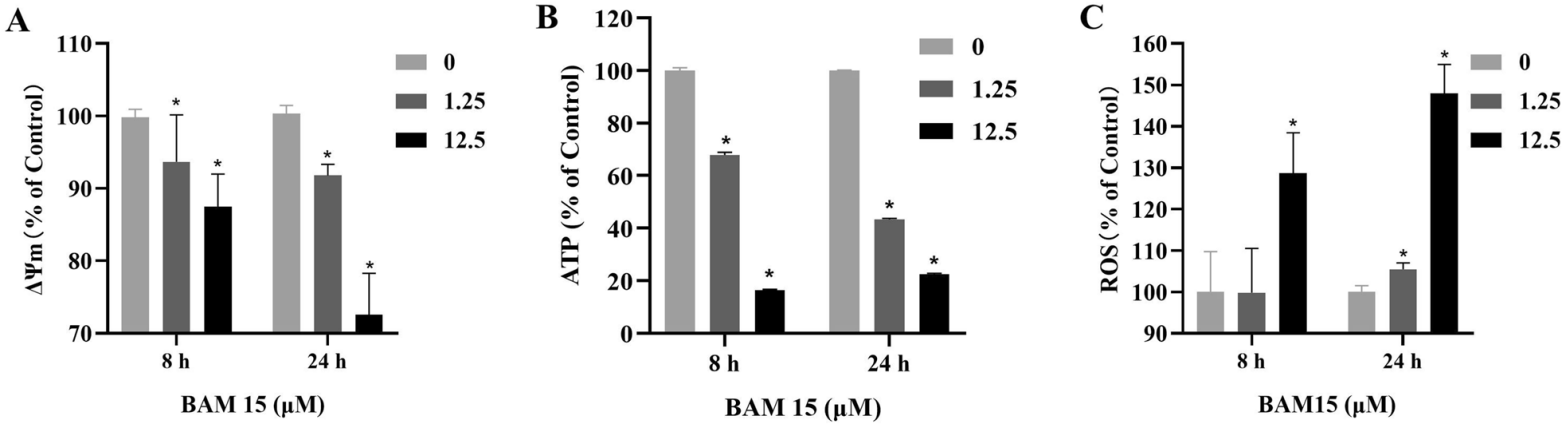
BAM15 induced a decrease in the mitochondrial membrane potential (**A**) and ATP concentration (**B**). BAM15 induced an increase in the ROS concentration (**C**). Data are presented as the mean value ± SD from three replicate experiments. *p < 0.01 compared with the control group

A MitoTracker Red CMXRos probe was used to evaluate the potential mitochondrial damage in tachyzoites treated with BAM15. Compared with the control group, treatment with BAM15 for 8 and 24 h resulted in a significant decrease in the Δ*Ψm* of *T. gondii* in a dose-dependent manner (*p* < 0.01; Fig. 7a). To further explore whether the effect of BAM15 on *T. gondii* is involved in energy transformation, we measured the ATP content of extracellular parasites after 8 and 24 h. ATP levels significantly decreased in the treatment groups compared with the control group (*p* < 0.01; Fig. 7b), confirming that the damage to *T. gondii* was related to mitochondrial oxidative phosphorylation. In particular, intracellular ROS levels increased in a dose-dependent manner after treatment with BAM15 for 8 and 24 h (*p* < 0.01; Fig. 7c).

### Anti-*T. gondii* activity of BAM15 in vivo

**Fig. 8:**
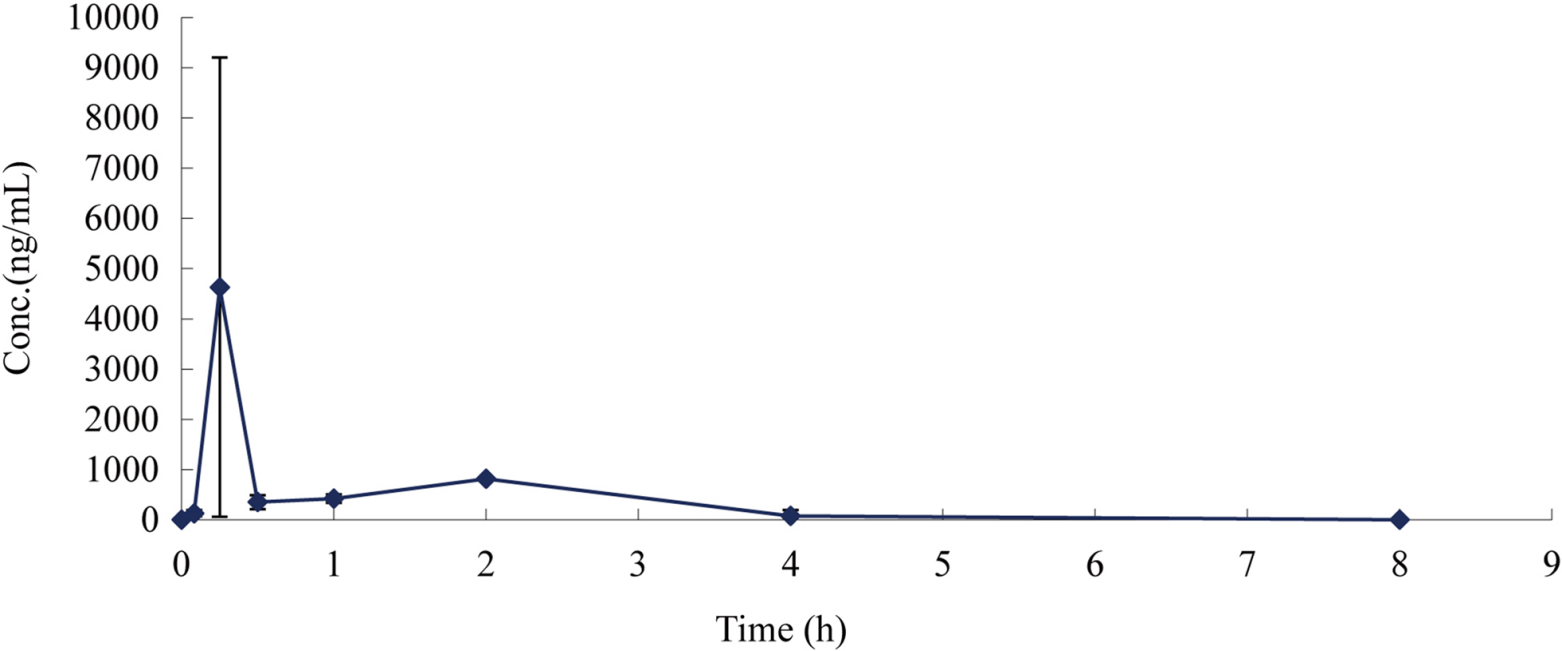
Mean plasma concentration-time profile after intraperitoneal injection of BAM15 into mice (*n* = *3*)

After intraperitoneal injection of 10 mg/kg BAM15 into mice, the plasma concentration at 24 h was measured. The non-compartment model was used to determine the main pharmacokinetic parameters of BAM15, as shown in Table 1. The *C*_max_ of BAM15 in mice was 4630 ± 4485 ng/ml (13.61 μM), *T*_max_ was 0.250 ± 0.433 h, AUC_0-t_ was 2724.355 ± 1750.565 h/ng·ml, and MRT_0-t_ was 1.233 ± 0.548 h. The mean plasma concentration-time profile after intraperitoneal injection of BAM15 into mice is shown in Fig. 8.

**Table 1:** Main pharmacokinetic parameters of BAM15 after intraperitoneal administration (*n* = *3*)

Parameters (units)	Mean ± SD
*K*_el_ (h^−1^)	0.744 ± 0.620
*T*_1/2_ (h)	0.932 ± 0.575
*T*_max_ (h)	0.250 ± 0.433
*C*_max_ (ng/ml)	4630 ± 4485
AUC_0-t_ (h/ng/ml)	2724.355 ± 1750.565
AUC_0-inf_ (h/ng/ml)	2823.166 ± 1965.769
MRT_0-t_ (h)	1.233 ± 0.548
MRT_0-inf_ (h)	1.376 ± 0.787

**Fig. 9:**
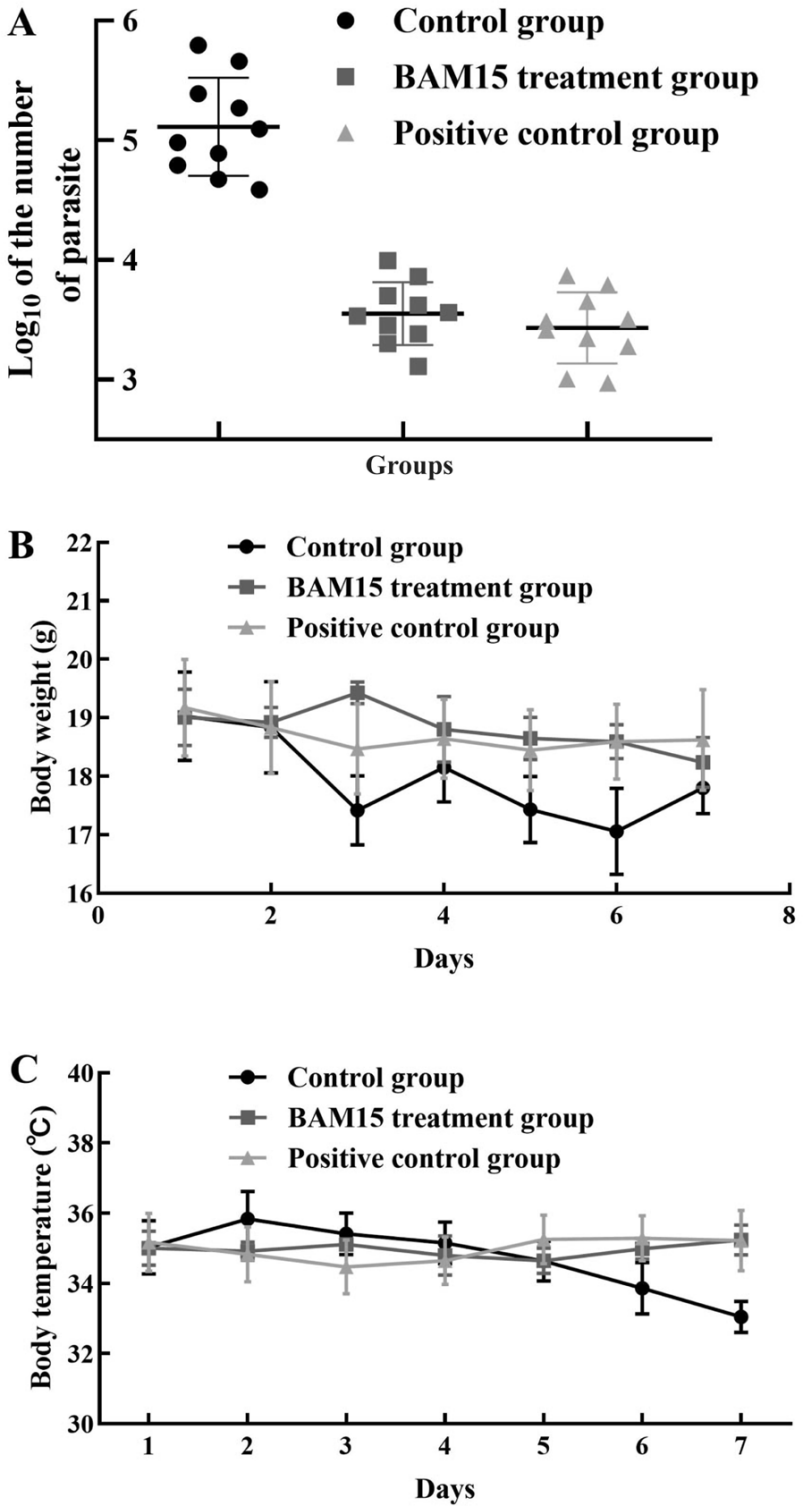
Administration of BAM15 decreased the *T. gondii* load in acutely infected mice (**A**). The body weight (**B**) and body temperature (**C**) are presented as the means ± SDs. *p < 0.01 compared with the parasite control

In the preliminary safety experiment to determine the side effects of BAM15, mice were injected with BAM15 intraperitoneally for > 7 days, and no obvious clinical toxicity was observed at any test drug dose during this period. At the same dose, BAM15 was used to treat mice infected with *T. gondii*. After treatment for 7 days, BAM15 significantly decreased the parasite burden in mouse peritoneum (Fig. 9a), slightly affecting the weight and temperature of the infected mice (Fig. 9b, c).

## Discussion

Toxoplasmosis is prevalent in humans and animals and is estimated to infect approximately 30–50% of the world's population [21]. However, the current treatment is limited because of toxic and side effects [22]. As the oxidative phosphorylation of mitochondria is vital for the survival of numerous eukaryotes, such as *T. gondii*, *T. gondii* has only one linked mitochondrion running through the interior, and destruction of mitochondrial function could affect important life processes in *T. gondii* [23]. Accordingly, *T. gondii* mitochondria are promising target organelles, and one underlying target is mitochondrial uncoupling. However, the main restriction on clinical application of mitochondrial uncouplers is their unnecessary targeting to the plasma membrane. As a mitochondrial uncoupler, BAM15 did not disturb plasma membrane transduction [24], and mild mitochondrial uncoupling protected cells from oxidative stress and mitochondrial damage [25], allowing BAM15 to control *T. gondii* infection with low cytotoxicity and resulting in an IC_50_ of 27.07 μM for Vero cells. This study further demonstrated that BAM15 has noteworthy inhibitory activity against *T. gondii* RH strain and PRU strain. In vitro anti-proliferative and anti-invasive assays have determined that BAM15 affected intracellular replication and invasion of *T. gondii* RH strain tachyzoites in a concentration-dependent manner under safe doses. The half-maximal inhibitory concentration of BAM15 on Vero cells was 22-fold higher than that on *T. gondii*, indicating that BAM15 had a high therapeutic index and a wide safe range. In addition, in the experiment of inducing PRU strains to form bradyzoite cysts in vitro, compared with the control group, the size and number of cysts were significantly reduced, and the shape of cysts was changed markedly, indicating that BAM15 had a significant inhibitory effect on the formation of cysts.

A previous study revealed that a mitochondrial uncoupler, niclosamide, at a dose of 240 mg/kg·bw, resulted in a 50% survival rate in infected mice because of poor solubility, absorption or systemic bioavailability [16]. In in vivo experiments, we established an infected mouse model to determine whether BAM15 has an *anti-T. gondii* effect on acute infection in vivo. Regarding the pharmacokinetic parameters, the *C*_max_ of BAM15 in mice was 4630 ± 4485 ng/ml, and the AUC_0-t_ was 2724.355 ± 1750.565 h/ng·ml, reaching the therapeutic concentration in vivo. Compared with the control, 10 mg/kg·bw BAM15 significantly reduced the parasite load in mouse ascites without obvious clinical toxicity. The body temperature and weight of the mice in the BAM15 group and positive drug treatment group were not significantly changed.

Previous studies have revealed that BAM15 could interfere with the energy metabolism of parasites by inhibiting the production of ATP, and one of the foremost advantages of BAM15 is characteristically depolarizing the membrane potential of mitochondria without affecting the plasma membrane structures of cells [26]. In the present study, TEM demonstrated that BAM15 induced mitochondrial swelling and degeneration in *T. gondii*, with little impact on the mitochondria of host cells. That is, BAM15 was very safe to host cells but harmful to *T. gondii* within the safe concentration range. Furthermore, there were dose-dependent decreases in the Δ*Ψm* and ATP level of *T. gondii*. The decrease in ATP production may lead to energy shortages in *T. gondii* and hinder the proliferation and invasion of tachyzoites. Moreover, the ROS content in tachyzoites increased after BAM15 treatment, disturbing the function of parasites and supporting the concept that the inhibitory effect of BAM15 on *T. gondii* may be related to mitochondrial oxidative phosphorylation. Nevertheless, its exact mechanism of action needs to be further explored.

## ﻿Conclusions

In brief, BAM15 exerted activity against *T. gondii* by inhibiting RH or PRU strain in vitro without host toxicity. In vivo experiments showed that BAM15 protected against the mice acutely infected with *T. gondii* and reduced the parasite burden in mouse ascites. TEM revealed that the anti-*T. gondii* mechanism of action of BAM15 interfered with the oxidative phosphorylation of *T. gondii* mitochondria, which was confirmed by decreased Δ*Ψm* and ATP levels or increased ROS levels. Therefore, BAM15 has the potential to be a safe and effective lead compound against *T. gondii*. The effects of BAM15 on animal models of infection with different strains of *T. gondii* and on cyst clearance in tissues will be further investigated.

## Data Availability

The data supporting the findings of the study must be available within the article and/or its supplementary materials, or deposited in a publicly available database. All data and materials generated or analyzed in this study have been provided in the article.
